# UniPR1331, a small molecule targeting Eph/ephrin interaction, prolongs survival in glioblastoma and potentiates the effect of antiangiogenic therapy in mice

**DOI:** 10.18632/oncotarget.25272

**Published:** 2018-05-11

**Authors:** Claudio Festuccia, Giovanni Luca Gravina, Carmine Giorgio, Andrea Mancini, Cristina Pellegrini, Alessandro Colapietro, Simona Delle Monache, Maria Giovanna Maturo, Roberta Sferra, Paola Chiodelli, Marco Rusnati, Annamaria Cantoni, Riccardo Castelli, Federica Vacondio, Alessio Lodola, Massimiliano Tognolini

**Affiliations:** ^1^ Department of Biotechnological and Applied Clinical Sciences, University of L'Aquila, 67100, L'Aquila, Italy; ^2^ Department of Food and Drug, University of Parma, 43124, Parma, Italy; ^3^ Department of Molecular and Translational Medicine, University of Brescia, 25123, Brescia, Italy; ^4^ Department of Veterinary Sciences, University of Parma, 43100, Parma, Italy

**Keywords:** glioblastoma, Eph/ephrin, angiogenesis, PPI-i, UniPR1331

## Abstract

Glioblastoma multiforme (GBM) is the most malignant brain tumor, showing high resistance to standard therapeutic approaches that combine surgery, radiotherapy, and chemotherapy. As opposed to healthy tissues, EphA2 has been found highly expressed in specimens of glioblastoma, and increased expression of EphA2 has been shown to correlate with poor survival rates. Accordingly, agents blocking Eph receptor activity could represent a new therapeutic approach. Herein, we demonstrate that UniPR1331, a pan Eph receptor antagonist, possesses significant *in vivo* anti-angiogenic and anti-vasculogenic properties which lead to a significant anti-tumor activity in xenograft and orthotopic models of GBM. UniPR1331 halved the final volume of tumors when tested in xenografts (p<0.01) and enhanced the disease-free survival of treated animals in the orthotopic models of GBM both by using U87MG cells (40 vs 24 days of control, p<0.05) or TPC8 cells (52 vs 16 days, p<0.01). Further, the association of UniPR1331 with the anti-VEGF antibody Bevacizumab significantly increased the efficacy of both monotherapies in all tested models. Overall, our data promote UniPR1331 as a novel tool for tackling GBM.

## INTRODUCTION

Glioblastoma multiforme (GBM), the most malignant brain tumor, shows high resistance to standard treatments consisting of different approaches that include surgery, radiotherapy and chemotherapy [1]. GBM shows a median overall survival (OS) of 14.6 months for newly diagnosed cases [2]. Failure of standard chemo/radiotherapy is attributed to multiple factors, such as microenvironment protection, *de novo* and/or acquired tumor resistance, limitations in drug delivery, increased angiogenesis and/or vasculogenic mimicry (VM), and presence of glioma stem cells (GSCs) [3].

Eph receptors are the largest subfamily of RTKs, with 16 known members. They are divided into “A” and “B” sub-classes, and they are activated by membrane proteins known as ephrins (Eph family receptor interacting proteins) [4]. These receptors are activated upon binding with their cognate ephrin ligands, which induce receptor clustering, followed by internalization and degradation. Eph receptors and their corresponding ligands play critical functions during early embryogenesis and development [5]. Recently, few Eph receptors along with their ligands have been implicated in the insurgence of several malignancies including GBM [6]. Indeed, the expression of EphA and EphB receptors in GBM has been correlated with poor prognosis [7]. Recent findings have proposed that EphA2 and EphA3 not only sustain the survival of GBM primary lines but also promote the renewal of tumor-propagating cells with stem-like characteristics (TPC). In these cells, both the receptors maintain an undifferentiated, self-renewing tumor population limiting MAPK signaling. In fact sustained ERK1/2 signaling leads to differentiation and reduces the proliferation and tumorsphere-forming capacity of GBM cells, whilst a transient ERK1/2 signaling keeps GBM cells in a stem-like state [8]. Additionally, Eph/ephrin signaling has been suggested to be a controlling factor in vasculomimicry (VM) [9] and tumor angiogenesis [10] where EphA2 emerged as a pivotal driver. Moreover, it has been recently demonstrated that Eph receptors activation through ephrin-B2 has a prominent role in perivascular invasion and in vascular co-option on GSCs [11].

These premises prompted several research groups to search for new antitumor strategies based on the exploitation of the Eph/ephrin system. These drug discovery expeditions allowed to identify ATP-mimicking agents targeting Eph kinase domain or other pharmacological tools including peptides, proteins, antibodies and small molecules, targeting Eph/ephrin binding interface [12]. We actively contributed to this field discovering and optimizing several protein-protein interaction (PPI) inhibitors capable of preventing Eph-ephrin interaction [13–15] and including the first orally bioavailable Eph antagonist UniPR1331 [16]. UniPR1331 targets the ectodomain of EphA2 with a steady-state affinity constant (K_D_) of 3.4 μM, dose-dependently blocked EphA2 phosphorylation in human umbilical vein endothelial cells (HUVEC) and reduced their ability to form blood vessels with an IC_50_ of 2.9 μM [16]. The compound inhibited the interaction of ephrin-A1 with all the EphA kinases and ephrin-B1 with all the EphB kinases [16] acting as a pan-Eph/ephrin inhibitor. Considering the primary role of the Eph/ephrin system in GBM and the availability of a compound able to specifically target the Eph receptors, we examined whether UniPR1331 was capable of inhibiting GBM growth *in vivo*. Since anti-angiogenic compounds targeting endothelial cells have been considered for treatment of recurrent GBM [17] but their clinical benefit was unsatisfactory [18, 19], we decided to test the association of UniPR1331 with Bevacizumab, a specific and selective inhibitor of VEGF-VEGFR interaction.

## RESULTS

### UniPR1331 inhibits *in vitro* and *in vivo* angiogenesis

**Figure 1:**
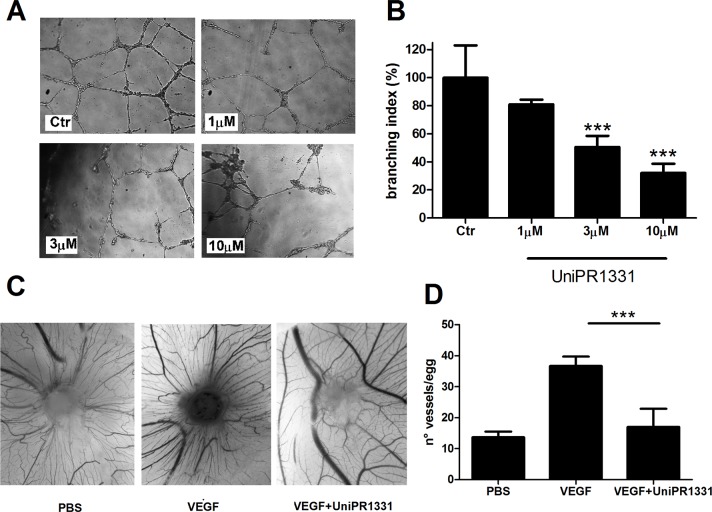
Effect of UniPR1331 on angiogenesis **(A)** representative pictures at 10x magnification. **(B)** Quantification of HBMVE cells tube formation as branching index analysis. **(C)** *in vivo* angiogenic assay. CAMs were implanted with alginate plugs adsorbed with or without VEGF and the compound. After 72 h the angiogenic response was scored. Representative photographs of CAMs incubated with/without VEGF in the absence or in the presence of 10μM UniPR1331. **(D)** Count of the number of vessels/egg in presence of VEGF (positive control) and after inhibition with 10 μM UniPR1331. Data are the mean ± SEM of 3 independent experiments ^***^: p<0.001, one-way ANOVA followed by Dunnett's post-hoc test.

UniPR1331 dose-dependently decreased *in vitro* tube formation of HUVE cells [16] and, here, we demonstrated that the compound reduced the phosphorylation of EphA2 receptor without modifying EphA2 expression after 16 hours (Supplementary Figure 1 and Supplementary Figure 2). The compound was tested on human brain microvascular endothelial cells (HBMVEC) and in the chick chorioallantoic membrane (CAM) assay, *in vivo*, in presence of soluble factors stimulating the proliferation of primitive vessels and their differentiation to a functional arteriovenous system. The compound blocked the tube formation of HBMVEC in a concentration-dependent manner, with an IC_50_ of 3.9μM (Figure 1A and 1B), consistent with its inhibitory potency of 2.9 μM on EphA2-ephrin-A1 and EphB4-ephrin-B1 displacement assays [16]. Anti-angiogenic activity of UniPR1331 was confirmed *in vivo* in the CAM assay, where it dramatically inhibited vessel formation induced by VEGF_165_ (Figure 1C, 1D), without a direct action on VEGFR2 as UniPR1331 did not interfere with the kinase activity of VEGFR2 (Supplementary Table 1). Beside the effect on vasculature cells, UniPR1331 inhibited ephrin-A1 induced EphA2 phosphorylation decreasing EphA2 expression after 24 hours on U87MG cells (Supplementary Figure 1, Supplementary Figure 2), without interfering with the enzymatic activity of EphA2 (Supplementary Table 1).

### UniPR1331 selectively targets Eph/ephrin system

The specificity of UniPR1331 for the Eph-ephrin system was evaluated by testing the compound on several targets promoting tumor angiogenesis (ICAM-1, PDGFR, and FGFR) and proliferation (TGF-β, EGFR). Since GBM growth is also regulated by the LXR-cholesterol axis [20], we asked whether UniPR1331 may display any effect on this system. When tested up to 10 μM, UniPR1331 did not modify the activity of any of these receptors (Supplementary Table 1). Finally, as the structure of UniPR1331 suggested connections with compounds targeting receptors or enzyme involved in metabolism of glucose, we tested it on TGR5, PPAR-γ, GLP-1 and DPP-IV (Supplementary Table 1). Again, UniPR1331 failed to affect the activity of these targets up to 10 μM.

### UniPR1331 inhibits GBM growth and extends the time to progression *in vivo* compared to the control: subcutaneous xenograft model

**Figure 2:**
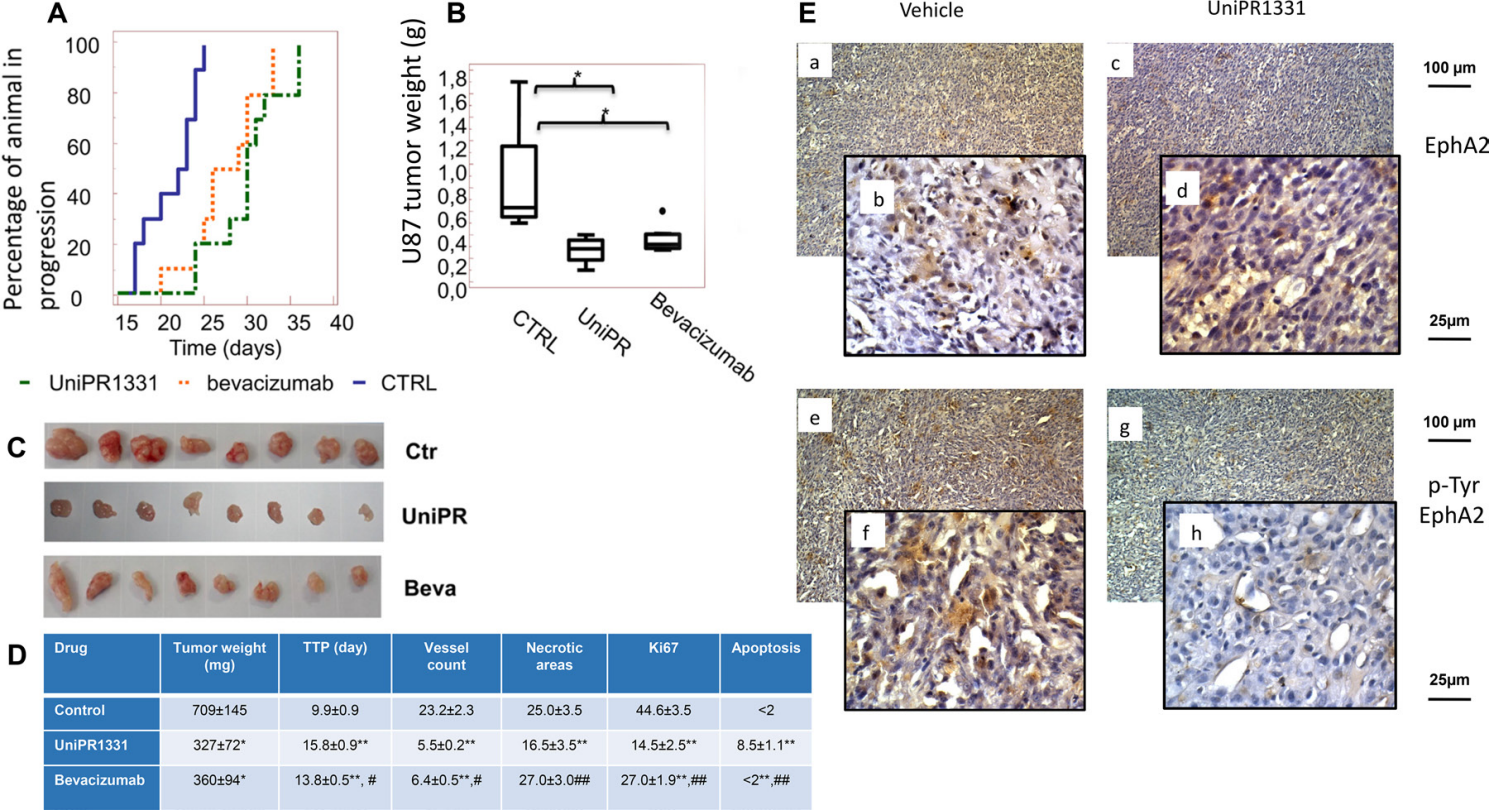
UniPR1331 blocks *in vivo* U87MG growth CD1 nude mice were subcutaneously injected with U87MG tumor cells and orally treated with 30 mg/kg UniPR1331, five days a week for five weeks. Tumor growth was assessed two times a week by measuring tumor diameters with a Vernier caliper. Bevacizumab (4 mg/kg iv every 4 days) was used as reference compound. **(A)** Kaplan-Meier curves generated for the comparisons of Bevacizumab, UniPR1331 and ctr. **(B)** Comparisons amongst treatments in terms of tumor weight, **(C)** Pictures of xenografted tumors. **(D)** Table reporting multiple parameters of the experiment. **(E)** Immunostaining performed on U87MG xenograft tissues collected from Vehicle-treated and UniPR1331-treated animals on total EphA2 and phospho-tyr-EphA2 expression ^*^, ^**^ p<0,05 or p<0.01 vs controls ^#^, ^##^ p<0,05 or p<0.01 UniPR1331 vs Bevacizumab, n=10.

UniPR1331 was tested *in vivo* for its ability to inhibit the tumorigenic activity of U87MG cells following oral administration in nude mice at 30 mg/kg os (five days a week) and the activity was compared with the standard anti-angiogenic drug Bevacizumab (4 mg/kg iv every 4 days) which inhibits VEGF receptors activation by binding to VEGF. To this aim, U87MG glioblastoma cells were subcutaneously injected and animals were randomly distributed in vehicle-, UniPR1331- or Bevacizumab-treated groups when tumors approximately reached 200mm^3^ volume. UniPR1331 exerted a significant inhibitory effect on the growth of human tumor grafts, compared to the control and showed an activity comparable to Bevacizumab (Figure 2). The treatment with UniPR1331 significantly increased the time to progression (TTP) from 9.9 to 15.8 days (Figure 2A, 2D) and halved the final tumor weight from 709±145 mg to 327±72 mg (Figure 2B, 2C, 2D). Moreover, the proliferation index Ki67 was reduced from 44.6% to 14.5%, the apoptotic cells increased from <2% to 8.5% and the vessel count dramatically decreased from 23.2 to 5.5. Similar results on TTP, tumor weight, and vessel count were obtained with Bevacizumab, whilst no effect was detected on apoptosis suggesting that, as opposed to Bevacizumab, UniPR1331 does not solely act as an antiangiogenic drug (Figure 2). It is worth note that toxicological studies showed that the treatment with UniPR1331 did not result in major adverse effects with the exception of a low platelet count of 207×10^3^/μl and minor adaptive changes in liver and kidney (Supplementary Table 2).

Immunohistochemical evaluation of EphA2 (Figure 2E, panels a, b) showed that this receptor stained U87MG tumor cells and endothelial cells (panel b) in about 40% of the tumor area (as shown in the low magnification 100x picture, panel a with a SI score of 6). Treatment with UniPR1331 reduced EphA2 immunostaining (Figure 2E panels c, d) to about 20% of tumor area (SI score=4). Further analyses showed that EphA2 is basally phosphorylated (on Tyrosine) in about 30% of tumor areas with a strong immunostaining (Figure 2E panel e, f with a SI=6). Treatment with UniPR1331 significantly reduced the immunostaining for p-Tyr-EphA2 (Figure 2E panels g, h, with a SI=2). A moderated staining was yet present in endothelial vessels (panel h) but not in vasculogenic mimetic vessels.

### Combination between Bevacizumab and UniPR1331 overcomes the limits of anti-VEGF based treatment in GBM xenografts

**Figure 3:**
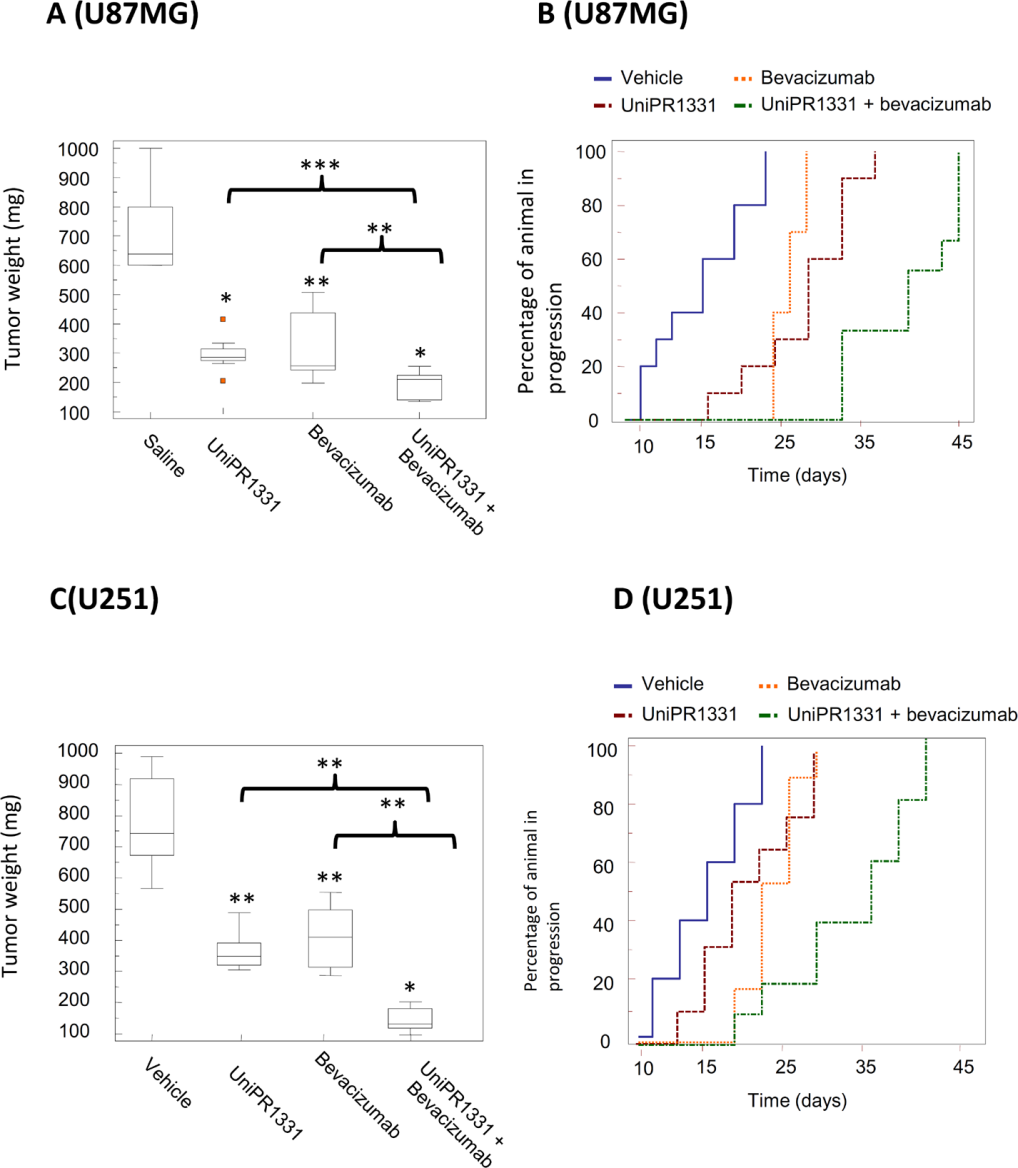
Combination between UniPR1331 and bevacizumab in GBM grafts CD1 nude mice were subcutaneously injected with either U87MG or U251MG tumor cells and treated with 30 mg/kg UniPR1331 os 5 days a week, 4 mg/kg Bevacizumab iv every 4 days or their combination for 30 days. Tumor growth was assessed two times a week by measuring tumor diameters with a Vernier caliper. **(A)** Comparisons amongst treatments in terms of tumor weight in U87MG grafts. **(B)** Kaplan-Meier curves were generated for the comparisons of control (ctr)- Bevacizumab- UniPR1331- and combination of Bevacizumab plus UniPR1331 in experimentation with U87MG cells. **(C)** Comparisons amongst treatments in terms of tumor weight in U251 grafts. **(D)** Kaplan-Meier curves were generated for the comparisons of control (ctr)- Bevacizumab- UniPR1331- and combination of Bevacizumab plus UniPR1331 in experimentation with U251MG cells. n=10.

We next tested the association of UniPR1331 (30 mg/kg os qd) and Bevacizumab (4 mg/kg iv every 4 days) on mice xenografts to assess its antitumor effect *in vivo* with U87MG and U251MG cells. In our experimental conditions, UniPR1331 significantly increased the efficacy of Bevacizumab, further reducing tumor growth. For instance, U251MG untreated tumors reached a final weight of 839 mg, whereas xenograft treated with UniPR1331 plus Bevacizumab reached a final weight of 157 mg vs 311 mg of UniPR1331 alone and 345 mg of Bevacizumab monotherapy. Association of UniPR1331 with Bevacizumab reduced tumor weights of 75% and 81% for U87MG and U251MG cells, respectively. The shrinkage of the tumor mass induced by oral administration of UniPR1331 in combination with Bevacizumab, when compared to control, resulted in a significant increase of the time-to-progression (TTP) of about 128% and 106% with U87MG and U251MG, respectively (Figure 3 and Supplementary Table 3–6). The association of UniPR1331 with Bevacizumab led to a dramatic increase of apoptotic index, a major decrease of ki67 and a further reduction of vessel count when compared with monotherapy alone, both with U87MG and U251MG grafts, suggesting the interference with multiple cancer pathways (Supplementary Table 3 and Supplementary Table 5).

**Figure 4:**
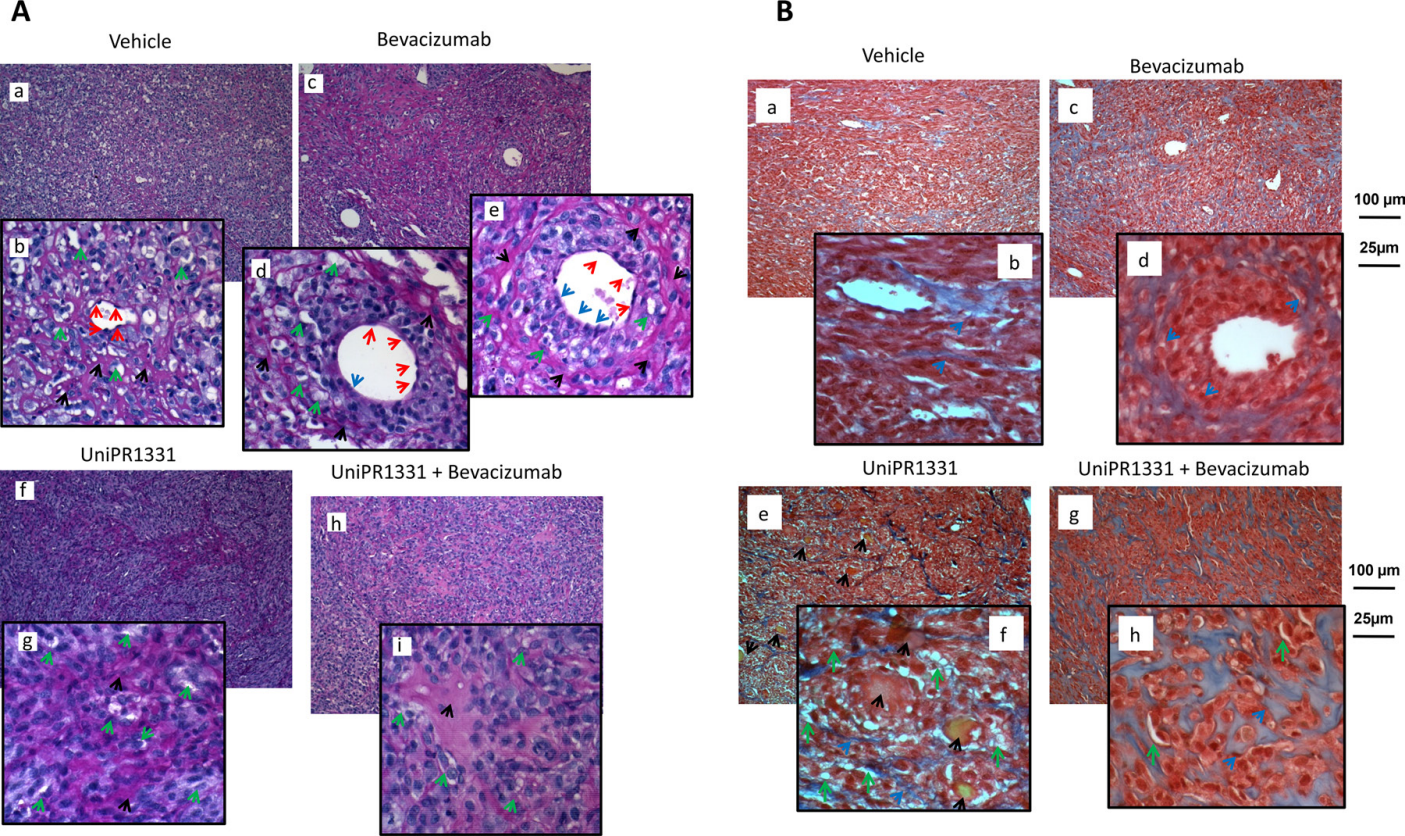
*In vivo* VM and angiogenesis from U87MG tumors **(A)** Periodic Acid-Schiff (PAS) staining: glycols are oxidized to aldehydes with subsequent formation of pararosaniline adducts which are stained in pink/red (black arrows). PAS stains the basal membrane underlying the vascular endothelium and delimits vasculo-mimetic structure. Subpanels a, b (vehicle treated animals) show main and detailed framework with endothelial cells (red arrows) delimiting a central small-sized blood capillary. Small vasculo-mimetic micro-capillaries (green arrows) are distributed uniformly in the tumor parenchyma. Subpanels c-e show PAS staining in Bevacizumab treated animals in which we have reduction of vessel number (subpanel c) and a switch from angiogenesis to vasculogenic mimicry starting from single medium-sized vessels (subpanel e blue arrows). We analyzed the two largest vessels in detail (subpanels d, e). Subpanels f, g: PAS staining in UniPR1331-treated animals showing a significant reduction in the number of medium/large-sized vessels as well as of vascular lacunae. Subpanels h, i: The reduced vascularization of the tissues was caused by UniPR1331 alone or co-administered with Bevacizumab. **(B)** Trichromic staining performed in Vehicle (subpanels a, b) Bevacizumab (subpanels c, d), UniPR1331 (subpanels e, f) and combination Bevacizumab plus UniPR1331 (subpanels g, h). Tumor areas rich in collagen fibers (stained in blue) indicating massive fibrosis colonized and eventually replaced vascular parenchyma of U87MG xenografts mainly in the combination treatment. In addition, in UniPR1331 treated tumors, Masson Trichrome staining highlighted the presence of deposits yellow/orange probably as blood clots following venous thrombi (black arrows).

Periodic Acid-Schiff (PAS) staining on xenograft histological specimens showed that angiogenesis and vasculogenic mimicry are equally distributed in untreated U87MG xenografts (Figure 4A panels a, b). Histological slides from Bevacizumab treated animals, showed a significant reduction of vessel number (Figure 4A, panel c) along with a switch from angiogenesis to vasculogenic mimicry starting from single medium-sized vessels. In Figure 4A (panel c), 4 medium-sized vessels are reported and we focused our attention on the largest ones. In the left one (panel d), the endothelial cells (red arrows) delimitate nearly the majority of the vascular wall. In a very little portion of it a loss of the endothelial cells continuity was observed (blue arrow) with two tumor cells oriented towards the lumen of the vessel. This phenomenon was more evident in the right vessel where about 50% of the vascular wall was without endothelium and composed by forming a palisade-like structure (Figure 4A, panel e). In this portion of the vessel, tumor cell layers were placed parallel to each other to give hardness to the vascular structure. The structure of the entire vessel was straightened by a dense PAS-positive connective matrix (black arrows). In the “vasculo-mimetic” region of the wall, a series of small vascular lacunae (micro-capillaries, green arrows) were also present. The number of these structures is considerably higher in this PAS positive-enclosed area (about 20) compared to those formed in the “angiogenic” portion of the vessel (about 7). Altogether, this analysis confirmed that Bevacizumab caused endothelial cell death but at the same time induced vasculogenic mimicry with several tumor cells engaged in the formation of vessel walls.

Administration of UniPR1331 resulted in a sensitive reduction of medium/large-sized vessels as well as of vascular lacunae. In these tissues, vascular lacunae are distribute in portions rich in PAS-positive regions (Figure 4A, panels f, g). This condition was increased when UniPR1331 was co-administered with Bevacizumab (Figure 4A, panels h, i) as this association abolished the Bevacizumab-induced vasculomimicry. In Figure 4A (panels f, g) wide non-vascularized areas were found in UniPR1331-treated tumors and they were significantly increased when Bevacizumab and UniPR1331 were combined (panels h, i). Altogether, this data suggests a possible fibrotic reaction. For this reason the trichromic staining was performed (Figure 4B). Wide areas rich in collagen fibers (stained in blue) indicate the presence of massive fibrosis (Figure 4B). In particular collagen fibers were dispersed in the parenchyma of untreated tumors (panels a and b) surrounding the vasculo-mimetic vessels in Bevacizumab treated specimens (panel c and d, blue arrows) and areas rich in vascular lacunae in UniPR1331 treated tumors (panel e and f). In addition, in UniPR1331 treated tumors, Masson Trichrome staining highlighted the presence of yellow/orange deposits probably as blood clots following venous thrombi (black arrows).

### Orthotopic intracranial model with U87MG cells and pharmacokinetic

**Figure 5:**
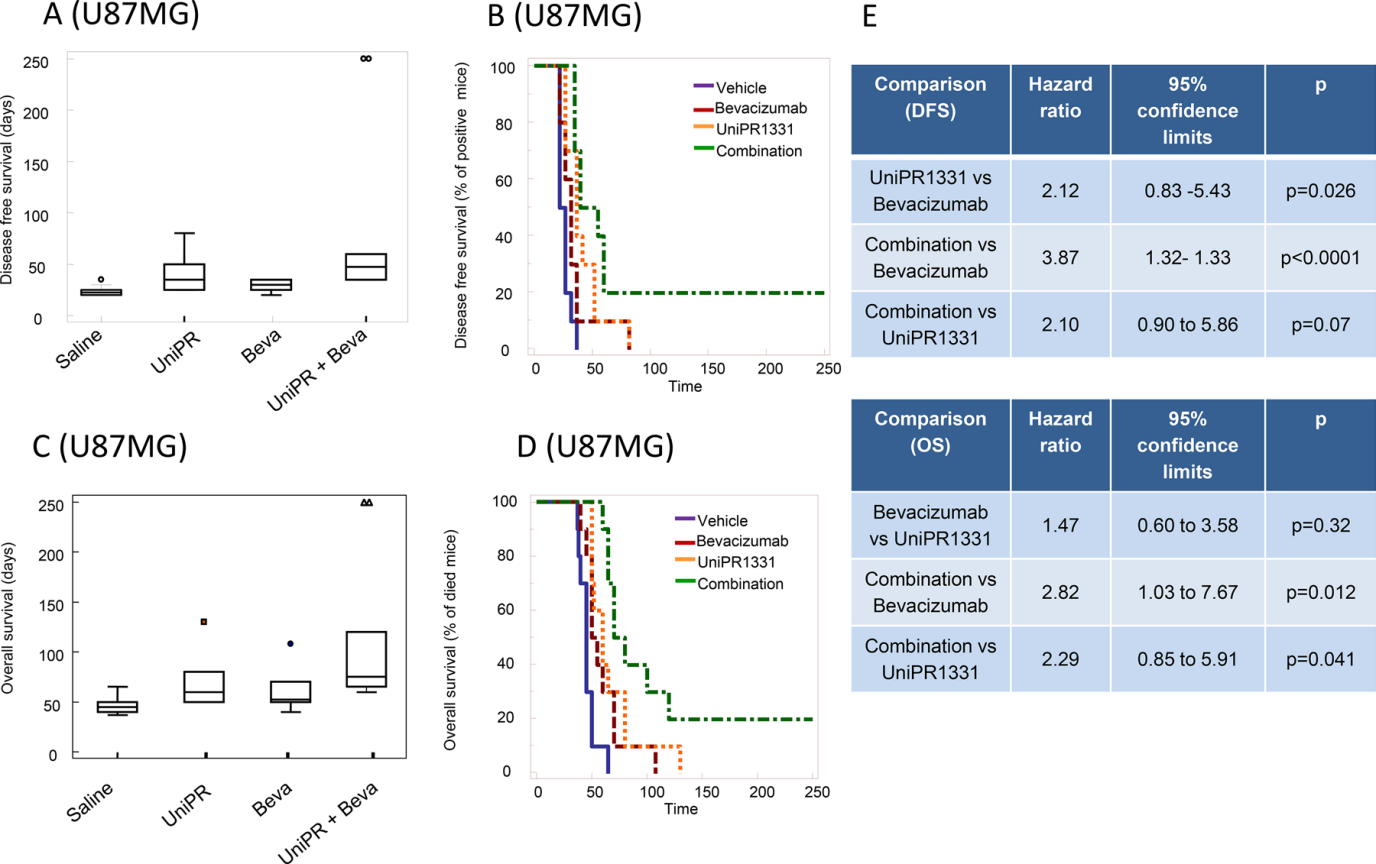
Combination between bevacizumab and UniPR1331 in U87MG orthotopic brain model CD1 nude mice were intra brain injected with luciferase transfected U87MG cells and treated with 30 mg/kg UniPR1331 os 5 days a week, 4 mg/kg Bevacizumab iv every 4 days or their combination. **(A)** Comparison amongst treatments in terms of tumor appearance or disease free survival (DFS). **(B)** Kaplan-Meier curves were generated for the comparisons of control (vehicle)- Bevacizumab- UniPR1331- and combination of Bevacizumab plus UniPR1331 in terms of disease free survival. **(C)** Comparisons amongst treatments in terms of overall survival. **(D)** Kaplan-Meier curves were generated for the comparisons of control (vehicle)- Bevacizumab- UniPR1331- and combination of Bevacizumab plus UniPR1331 in terms of overall survival. **(E)** Statistical analysis: comparison of Kaplan Meier curves for UniPR1331 alone or in combination with Bevacizumab. n=10.

Before performing *in vivo* experiments using orthotopic cancer models with luciferase-expressing cells, we assessed the ability of UniPR1331 to access central nervous system (CNS) in healthy mice. The compound was administered 30 mg/kg os and it reached a maximal plasma concentration (C_max_) of 850 nM after 30 minutes (t_max_). Measurements by HPLC/MS of brain homogenates at selected time points (15’, 30’, 1h, 4h) after oral administration of UniPR1331 confirmed that the compound crosses the brain-blood barrier reaching a C_max_ of nearly 100 nM at 30 minutes, with a plasma/brain concentration ratio close to 8, consistent with literature data [21] for compounds with similar lipophilicity (Supplementary Figure 3). Treatment with UniPR1331 (DFS= 40.0 ± 16.8 days) or Bevacizumab (DFS = 28.5 ± 5.8 days) for 30 days, significantly increased the disease-free survival (DFS) period when compared to control (24.0 ± 5.2 days). A dramatic improvement of DFS was observed when UniPR1331 was administered in combination with Bevacizumab (DFS = 88.00 ± 27.5 days) Figure 5A, 5B. Analysis of the Kaplan-Meier curves indicated that the combination between UniPR1331 and Bevacizumab was significantly more effective than the treatment with both drugs taken individually (Figure 5E). Similar results were obtained considering the overall survival: UniPR1331 (OS=67.7 ± 7.8 days) and Bevacizumab (OS=59.8 ± 6.2 days) significantly increased the OS when compared to control (OS=42.9 ± 2.7 days, p<0.05). A further improvement in the OS was observed when UniPR1331 was combined with Bevacizumab (OS=113.00 ± 20.6 days) (Figure 5C, 5D, 5E). These results suggest that the combination of UniPR1331 with Bevacizumab showed a cumulative effect on DFS and OS in differentiated U87MG cell model.

### Orthotopic intracranial model with tumor-propagating cells

**Figure 6:**
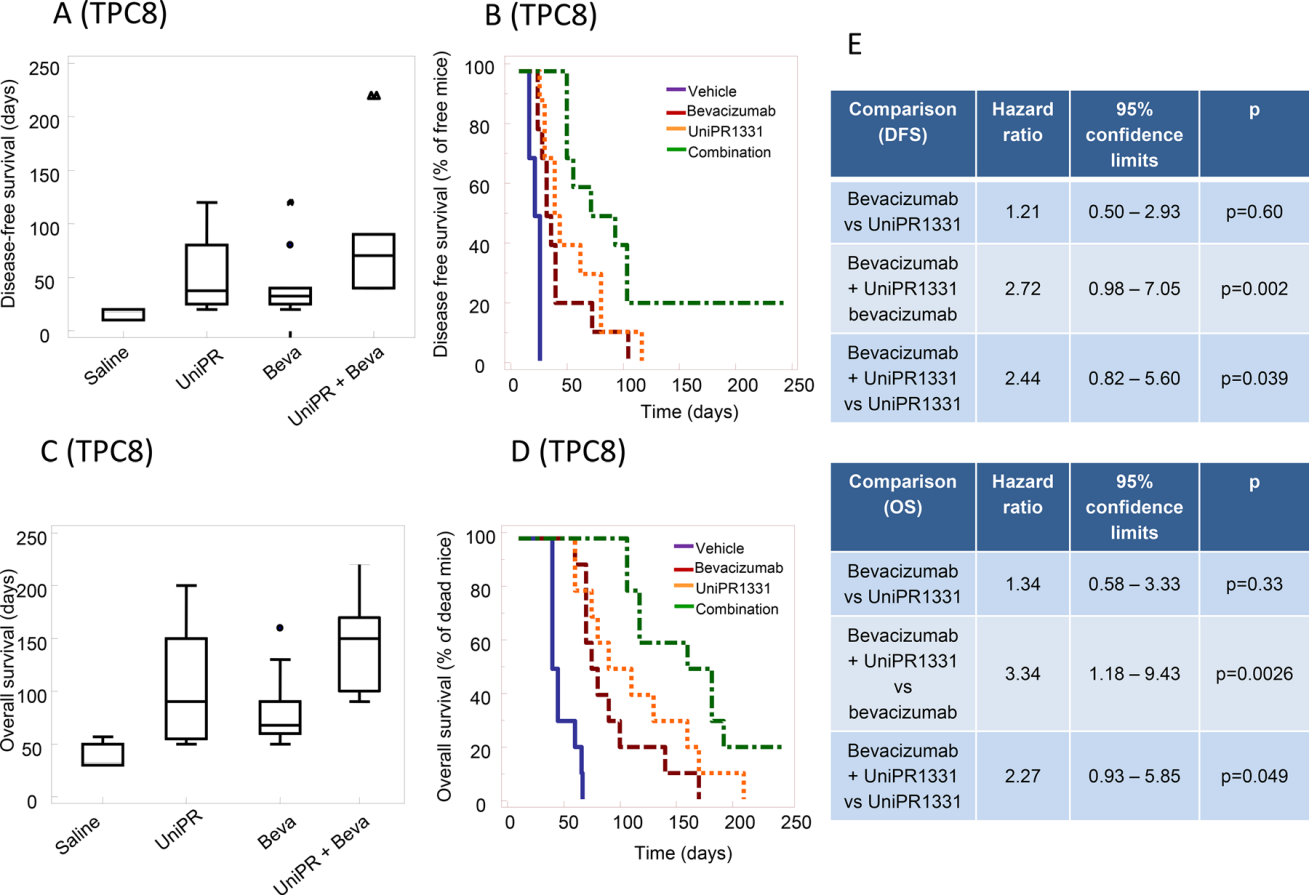
Combination between bevacizumab and UniPR1331 in TPC8 orthotopic brain model CD1 nude mice were intra brain injected with luciferase transfected TPC8 cells and treated with 30 mg/kg UniPR1331 os 5 days a week, 4 mg/kg Bevacizumab iv every 4 days or their combination for 30 days. Tumor growth was assessed two times a week by measuring tumor diameters with a Vernier caliper. **(A)** Comparisons amongst treatments in terms of disease free survival (DFS). **(B)** Kaplan-Meier curves were generated for the comparisons of control (ctr)- Bevacizumab- UniPR1331- and combination of Bevacizumab plus UniPR1331 in terms of disease free survival. **(C)** Comparisons amongst treatments in terms of overall survival. **(D)** Kaplan-Meier curves were generated for the comparisons of control (ctr)- Bevacizumab- UniPR1331- and combination of Bevacizumab plus UniPR1331 in terms of overall survival **(E)** Statistical analysis: comparison of Kaplan Meier curves for UniPR1331 alone or in combination with Bevacizumab. n=10.

Since EphA2, EphA3 and ephrin-B2 were described to regulate tumor-propagating cell self-renewal [8, 11, 22], luciferase-transfected glioma stem cells (TPC8 cell line) were injected into mouse brains. In these conditions, 30 days treatment with UniPR1331 (52.0 ± 10.2 days) and Bevacizumab (DFS=44.0 ± 10.0 days) significantly (p<0.005) increased the DFS period when compared to control (16.0 ± 1.4 days). Kaplan-Meier curves showed no significant differences amongst single monotherapies (Figure 6A, 6B, 6E) while the association between UniPR1331 and Bevacizumab (DFS=92.5 ± 22.2 days) displayed higher efficacy than treatments with both drugs taken individually. The increased lapse of tumor appearance was associated with a significant increase in median OS (Figure 6C-6D) of treated animals. The analysis of Kaplan-Meier curves indicates that Bevacizumab (OS=82.5 ± 3.6 days) and UniPR1331 (101.5 ± 16.5 days) were effective in the increase of OS of TPC8 bearing animals. UniPR1331 increased the efficacy of Bevacizumab both in terms of mean OS (145.0 ± 15.8 days, p<0.01) and in term of reduction of mice in progression (HR=3.34, p<0.01). This combination resulted also significantly more effective than UniPR1331 administered alone (HR=2.44, p<0.05 for DFS and HR=2.27, p<0.05 for OS) (Figure 6E).

### Effect of UniPR1331 on miRNA involved in blood vessel formation

**Figure 7:**
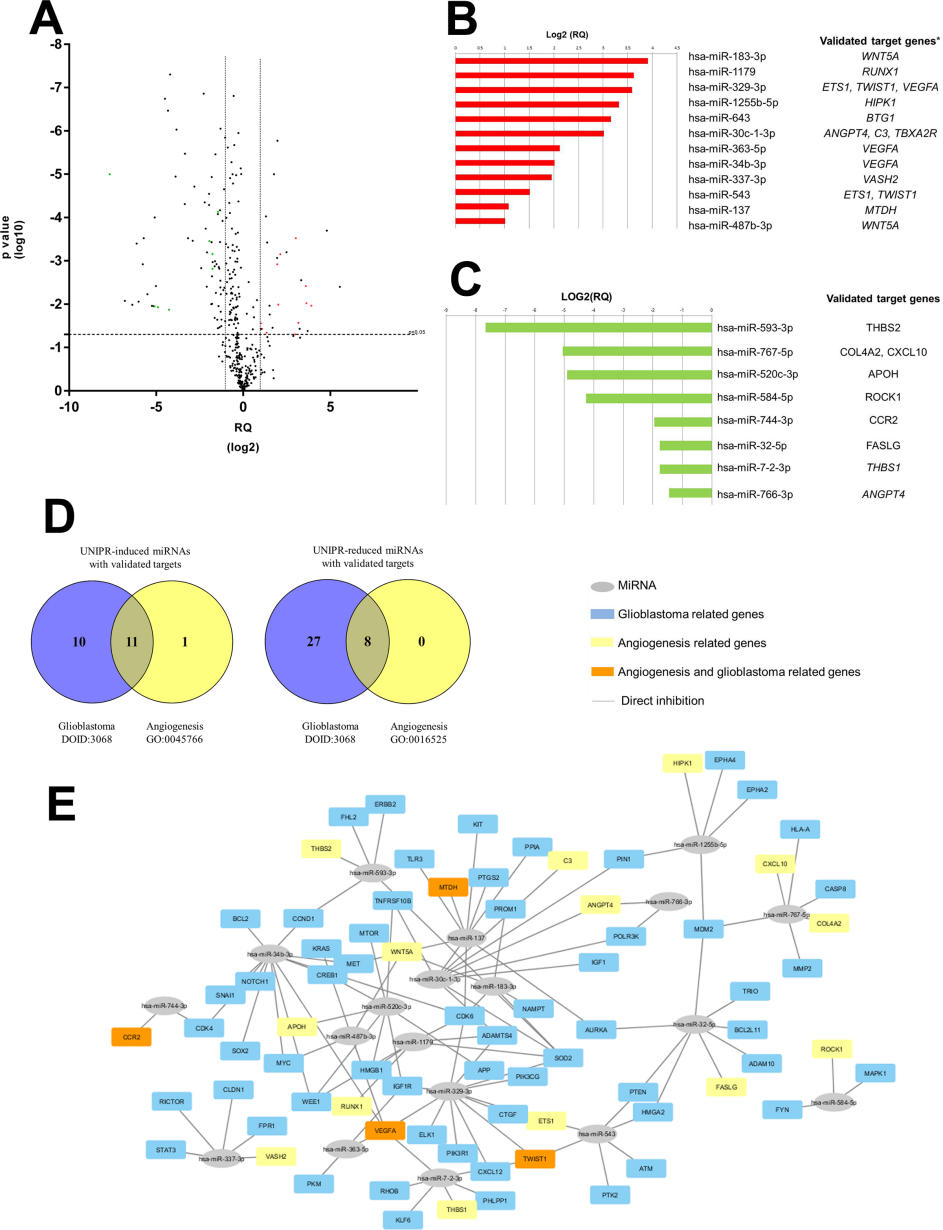
Identification of UniPR1331-deregulated miRNAs influencing the “cancer stem cell-regulated angiogenesis” in glioblastoma **(A)** Volcano plot representation for the global trend of UniPR1331-deregulated miRNAs. The green points represent down-regulated miRNAs that have validated targets in GO:0016525 term (negative regulation of angiogenesis); the red points represent up-regulated miRNAs that have validated targets in GO:0045766 ontology (positive regulation of angiogenesis), according miRWalk analysis. **(B** and **C)** Relative expression levels (log2[RQ]) of UniPR1331-induced and UniPR1331-reduced miRNAs with experimentally proven targeted genes, respectively. **(D)** Venn diagram shows the numbers of UniPR1331-deregulated miRNAs with validated targets influencing the angiogenesis reduction, the glioblastoma disease and their overlaps. **(E)** Cytoscape 3.4.0 software graphical visualization of miRNA-target interactions. The colors of each gene node indicate the annotated function of the gene.

We finally set to test whether UniPR1331 and Eph/ephrin targeting could interfere with protein expression of TPC8 cells. MiRNAs are small, non-coding RNAs that mediate post-transcriptional gene silencing. Since the recurrence in a resected and treated GBM is associated to cancer stem cell recruitment and growth, global miRNAs profile was assessed in TPC8 cells line following exposure to 10 μM UniPR1331 or DMSO 1% for 24 hr. A total of 184 mature miRNAs, including 35 UniPR1331-induced and 149 UniPR1331-reduced miRNAs, significantly (p<0.05) distinguished treated cells from those untreated. Twenty-seven miRNAs exhibited a significant >2-fold UniPR1331-induced-up-regulation (RQ>2) and 70 miRNAs showed <2-fold down-regulation (Figure 7A). The most highly expressed UniPR1331-induced miRNAs (RQ> 20) were hsa-miR-616-3p and hsa-miR-15a-3p whereas the most UniPR1331-reduced miRNAs (RQ < 0.02) were hsa-miR-593-3p, hsa-miR-144-5p, hsa-miR-501-3p, hsa-miR-326 and hsa-miR-199a-3p. On the basis of the Validate MiRWalk Module bioinformatics tool, used to identify the UniPR1331-deregulated miRNAs (targeting genes that have been proved experimentally), we selected 12 over-expressed and 8 down-expressed miRNAs able to silence genes involved in the angiogenesis (Figure 7B and 7C). Furthermore, we identify 21 UniPR1331-induced and 35 UniPR1331-reduced miRNAs targeting glioblastoma-related genes (DOID:3068 ontology) (Supplementary Tables 7 and 8), with 11 over-expressed and 8 down-expressed all influencing glioblastoma and promoting angiogenesis inhibition (Figure 7D and 7E). The prediction analysis revealed that 27 UniPR1331-induced miRNAs were significantly enriched for their binding sites within the genes associated with angiogenesis activation (gene ontology GO:0045766) and 38 UniPR1331-reduced miRNAs targeted genes associated with angiogenesis inhibition (gene ontology GO:0016525) (data not shown). According to MiRWalk prediction algorithms, 7 over-expressed and 19 down-expressed (Supplementary Table 9) miRNAs could significantly (p <0.01) acted to reduce angiogenesis process in UniPR1331-treated TPC8 cells. Some miRNA may be associated to VM.

## DISCUSSION

The overexpression of EphA2 in GBM tissues promotes glioma cell migration and invasion [23–26], while its activation concurs to the renewal of cancer stem cells [22, 27]. Considering that the Eph/ephrin system also regulates angiogenesis [10], antagonists of this system may elicit beneficial effects in the treatment of several cancer types including GBM. Different antibodies or recombinant proteins targeting Eph/ephrin system have been already used to exploit this cellular system in the search for novel cancer therapies. Amongst the available therapeutic options, the recombinant EphB4-HSA fusion protein [28], the anti-EphA2 monoclonal antibody DS-8895 (NCT02004717, NCT02252211), the antibody-drug conjugated PF-06647263 (a humanized monoclonal antibody against Ephrin-A4 linked to calicheamicin (NCT02078752)) and KB004, [29], are under clinical I or II studies for oncological therapies. These tools are capable of blocking both forward and reverse signaling; however their high specificity towards ephrin-Bs, EphA2 and EphA3 respectively, represents a potential weakness. In fact, signaling mediated by other Eph-ephrin pairs can lead to the activation of survival pathways overcoming the inhibition of the selected target. Kinase inhibitors (dasatinib, Jl-101, XL647) targeting the intracellular kinase domain of the Eph receptors are available but compared to other agents their use is hampered by limited efficacy considering that: 1 they are unable to prevent activation of reverse signal generated in the ephrin-expressing cells, 2. they suffer limited selectivity with insurgence of side effects associated to inhibition of other kinases (i.e., EGFR, VEGFR, SRC, KIT) [30] and 3. they could suffer a difficult access to the target which is localized in the cytosol of cancer cells. In this context, small molecules targeting Eph/ephrin interaction, such as UniPR1331, represent an alternative way to current therapeutic approaches based on recombinant proteins and kinase inhibitors, as they directly target the interaction amongst all the Ephs and ephrins blocking both the reverse and forward signaling [12].

Amongst all these targeting agents, the ephrin-B2 blocking scFv antibody fragment (B11) and the radiolabelled lutetium-177 monoclonal antibody IIIA4 were tested in animal models of GBM. The first strongly suppressed tumor growth of highly expressing ephrin-B2 GSCs orthotopically implanted in mice [11], the latter induced a dose-dependent tumor regression of U251MG and BAH1 cells subcutaneously xenografted mice [8]. Both the treatment showed no evident toxicity.

In this study, we tested for the first time the activity of a small molecule (UniPR1331) targeting protein-protein interaction which synthesis, chemical characterization and preliminary pharmacological investigations at an *in vitro* level were recently published [16]. To the best of our knowledge, UniPR1331 is the only orally bio-available small molecule that can be regarded as a genuine Eph/ephrin antagonist, as other small molecules reported to block Eph receptors (i.e. 2, 5-dimethyl-pyrrol-1-yl-benzoic acid, Rhyncophylline, Protocatechuic acid, Pyrogallol, Urolithin D, Epigallocatechin-3, 5-digallate) were recently demonstrated to be PAINS, compounds endowed with high reactivity and being able to unspecifically react with many biological targets [12, 31, 32]. UniPR1331 showed good tolerability since only a reduced platelet count, which was probably connected to the interference with hematopoietic stem/progenitor cells [33], was revealed in accordance with previous studies [8, 11]. However, future studies should be taken in account to evaluate reproductive/developmental toxicity since the Eph/ephrin system plays a primary role in embryo development [5].

UniPR1331 dramatically reduced GBM growth in two distinct xenograft models (based on U87MG and U251MG cells), leading to a significant increase of TTP. Biochemical and histological analysis indicated that the inhibition of GBM growth was due to a marked reduction of blood vessel formation, inhibition of VM and activation of apoptosis. In the same models, Bevacizumab showed similar efficacy blocking angiogenesis but it did not significantly modify cell proliferation or apoptosis. Notably, Bevacizumab did not lead to a full de-vascularization of the tumor as numerous medium-sized vessels were still present in the tumor parenchyma at the end of the treatment. These vessels were characterized by the presence of several GBM cells in the vascular wall where they replaced endothelial cells consistent with a VM process. The resulting palisade structures, formed by a majority of GBM cells, gave origin to numerous vascular lacunae, which being dispersed in the tumor parenchyma, may supply oxygen and nutrients to tumor cells far from the main vessel. VM was previously described as a survival mechanism adopted by the tumor tissues [34]. During VM, cancer stem-like cells [35] acquire the ability to form vessel-like networks mimicking the physiological vasculature. In conditions of hypoxia, due to impairment in angiogenesis, VM developed as a possible strategy to ensure blood supply [36] to cancer cells [37, 38]. Among the wide array of signaling systems implicated in VM, the Eph receptors have been proposed as novel and relevant players. The involvement of the Eph/ephrin system in VM has been recently proposed [39, 40] with their aberrant expression on GBM cells linked to perivascular invasion and vascular co-option [11, 22].

Consistent with this view, combination of UniPR1331 with Bevacizumab reduced angiogenesis and vasculomimicry leading to large avascular tumor areas in U87MG tumors. A fibrotic reaction was also observed with the use of UniPR1331, alone or in combination, suggesting that its effect modified the structure of the tumor parenchyma with areas featured by the lack of tumor cells. Since GBM is highly heterogeneous, we evaluated whether the activity of UniPR1331 alone or in combination could be observed in other GBM models. To this end, in addition to U87MG, U251 and TPC8 cells were used to generate heterotypic and orthotopic xenograft models. Consistent results were obtained in all the tumor models, with the combination able to improve the efficacy of Bevacizumab. In particular, a significant increase of DFS and OS was found when patient-derived glioblastoma propagating/undifferentiated/stem cells, TPC8, were injected in the brain of nude mice.

TPC8 cells were also used to identify molecular pathways that might be affected by use of UniPR1331 following the significant changes in miRNA expression. MiRNAs are small non-coding RNA molecules that act by base pairing to complementary regions of their mRNA targets, leading to mRNA degradation and/or inhibition of protein translation [41]. Remarkably, all but one of these miRNAs also targeted genes involved in glioblastoma pathogenesis, suggesting a further potential molecular explanation of the combined anti-angiogenic and anticancer activity of UniPR1331 observed in *in vivo* models.

As observed in Figure 7, 12 miRNAs were up-regulated by UniPR1331 administration resulting in the reduction of a series of gene products including VEGF-A (miR-329, miR-263 and mir-34b), WNT5A (miR-183, miR-487b), Twist1 (miR-183 and miR-329) and ANGPT4. Besides the well-known activity of VEGF-A on angiogenesis, Wnt5A signaling is emerging as a major mediator in cancer progression, regulating cancer cell invasion, metastasis and metabolism [42]. Twist1 upregulation or activation is involved in EMT, metastasis, angiogenesis, and is responsible for the “stemness” of cancer cells and the generation of drug resistance, gaining significance in cancer therapeutics [43]. Angiopoietin-4 is involved in glioblastoma progression by enhancing tumor angiogenesis and cell viability [44]. Despite reports that miR-26b inhibited the VM which processes is regulated by EphA2 [45] we have found no relevant difference in our experiments. Taken together our data indicate a complex network of interactions, where each mRNA can be regulated by multiple miRNAs and at one time, each miRNA can potentially target multiple genes involved in many facets of cancer progression. In this context, our work might not represent an exhaustive study investigating the functional activity of certain UniPR1331-deregulated miRNAs, but aims at providing further evidences that UniPR1331 inhibits GBM, by modulating different facets of cancer progression.

## MATERIALS AND METHODS

### Cell lines and cell cultures

All the materials for tissue culture were purchased from Euroclone. Human glioma cell lines U251MG and U87MG were originally obtained from American Type Culture Collection (ATCC, Rockville, MD). Luciferase transfected U87MG cells were kindly provided by Jari E. Heikkila, Department of Biochemistry and Pharmacy, Abo Akademi University, Turku, Finland. Cells were cultured at 37°C in 5% CO_2_ and were maintained as suggested. Patient-derived tumor-propagating cells (TPC8) were kindly provided by prof. A. Vescovi (Università Bicocca, Milano) and Dr L. Balconi (Stemgen, Milano) [22]. To confirm their stem-like cell nature immuno-fluorescence by confocal analyses and FACS determination were performed using Sox2, Nestin, CD44, oct3/4 and GFAP. TPC8 were cultured in serum-free DMEM/F-12 (Life Technologies) supplemented with EGF/basic FGF. hBMVEC (human brain microvascular endothelial cells) were kindly provided by Philip M. Cummins, School of Biotechnology, Dublin City University, Ireland. The endothelial cell line was routinely grown in complete endothelial cell medium (EGM™-Plus, Lonza Biologics, Slough, UK) containing heparin (0.75 U/ml), hydrocortisone (1 μg/ml), recombinant human epidermal growth factor (5 ng/ml), EndoGRO-LS Supplement (0.2%) and antibiotics. HUVEC (Life Technologies, Waltham, MA, USA) were maintained in MEM 200 supplemented with 1% penicillin-streptomycin solution, 1% fungizone solution, 2% low serum growth supplement and 10% FBS. To minimize the risk of working with misidentified and/or contaminated cell lines, the cells used in studies reported here were stocked at very low passages after initial receipt from the vendor to reduce the possibility of contaminated cell line stocks and used at < 20 subcultures. Periodically, DNA profiling by GenePrint® 10 System (Promega Corporation, Madison, WI, USA) was carried out to authenticate cell cultures.

### Chick-embryo chorioallantoic membrane (CAM) assay

The assay was performed as described [46]. Briefly, a window was opened in the eggshell of three-day-old fertilized chicken eggs. At day 11, alginate plugs containing VEGF-165 (4.5 pmoles/embryo) in the absence or in the presence of the compound (20 pmoles/embryo) were placed on the CAMs (8 embryos per group). At day 14, newly formed blood microvessels converging toward the implant were counted.

### Anatomo-pathological analysis

Anatomo-pathological analysis were performed on 10 male mice treated with UniPR1331 30 mg/kg os. Kidneys, spleen, thymus, testicles, accessory sexual glands, stomach, segments of duodenum and jejunum, heart, lungs and brain were collected from each mouse at the end of experimentation and samples were immediately fixed in 10% neutral buffered formalin. After paraffin embedding, 4-5 μm thick sections, were obtained with microtome (Leica), stained with Hematoxylin and Eosin (H&E) and Periodic acid-Schiff (PAS). Histological slides were examined with Nikon Eclipse E800 microscope (Nikon Corporation, Japan) using Nikon PLAN APO lenses. Sections were photographed at 4x, 10x, 20x and 40x (Nikon PLAN APO lenses) with Camera DIGITAL SIGHT DS-Fi1 (Nikon Corporation, made in Japan); pictures were acquired with DS Camera Control Unit DS-L2 (Nikon Corporation, Japan) and stored in USB device. Histological lesions were graded and classified based on the extension/distribution (focal, multifocal and diffuse) and severity (scant, mild, moderate, severe).

### Pharmacokinetic studies

UniPR1331 was suspended in 0.5% methylcellulose (10/90 v/v) and orally administered as a single gavage at 30 mg/kg to male mice. Each group consisted of at least three mice. Blood samples were collected via tail puncture at different time-points. Whole blood samples were centrifuged at 5000 rpm for 10 min, and the resulting plasma samples were stored at 20°C pending analysis. UniPR1331 was dosed in mouse plasma by HPLC-ESI-MS/MS using a Thermo Accela UHPLC gradient system coupled to a Thermo TSQ Quantum Access Max triple quadrupole mass spectrometer (Thermo Italia, Milan, Italy) equipped with a heated electrospray ionization (H-ESI) ion source. For assessing UniPR1331 CNS penetration mice brains were removed and homogenized (20% (w/v), wet tissue) in PBS buffer (pH 7.4). The compound was quantified using the same approach described for blood analysis, but reducing the number of time points to 5. Xcalibur 2.1 software (Thermo Italia, Milan, Italy) was used for sample injection, peaks integration, and plasma level quantification.

### *In vivo* GBM models: xenograft model

Female CD1-nu/nu mice, at 6 weeks of age, were purchased from Charles River (Milan, Italy) and maintained under EC guidelines (2010/63/UE and DL 26/2014 for the use of laboratory animals). All mice received subcutaneous flank injections of 1 × 10^6^ U87MG and U251MG. Tumor growth was assessed bi-weekly by measuring tumor diameters with a Vernier caliper. Tumor weight was calculated according to the formula: TW (mg) = tumor volume (mm^3^) = d^2^ x D/2, where d and D are the shortest and longest diameters, respectively. The effects of the treatments were examined as previously described [48]. At about 20 days after the tumor injection, mice with tumor volumes of 200 mm^3^ were randomized to receive vehicle, Bevacizumab (4 mg/kg iv every 4 days), or UniPR1331 (30 mg/kg po qd), or combinations of Bevacizumab with UniPR1331. The duration of treatments was 30 days when control/untreated tumors reached critical volumes for animal welfare laws. Treated and untreated animals were, therefore, sacrificed by carbon dioxide inhalation and tumors were subsequently removed surgically at the same time. This was necessary for the biochemical and histological analyses. A part of the tumor was, indeed, directly frozen in liquid nitrogen for protein analysis and the other part was fixed in paraformaldehyde overnight for immunohistochemical analyses. The following parameters were used to quantify the antitumor effects of different treatments as previously described [47]: (1) tumor volume, measured throughout the experiment, (2) tumor weight, measured at the end of experiment; (3) tumor progression (TP) defined as an increase of 100% of tumor volume (named also doubling time) with respect to baseline in preclinical model in agreement with Reynold et al [48]. The use of TTP over tumor growth curves was chosen to reflect the pharmacological efficacy assessment in humans and to reduce the variability given by volumetric measures due to differences of engraftment of the tumor cells as well as the individual variability [47]. Combination index of dual administrations was calculated accordingly to Bruzzese et al [49].

### Orthotopic intra-brain model

Nude mice were inoculated intra-cerebrally as follows [50]. Animals were anesthetized with 100 mg/kg ketamine, 15 mg/kg xylazine. The surgical zone was swabbed with Betadine solution, the eyes coated with Lacri-lube. The head was fixed in a stereotactic frame (mouse stereotaxic instrument, Stoelting Europe, Dublin, Ireland) and a midline scalp incision was made. A small hole was made at 1.0 mm anterior and 2 mm lateral to the exposed bregma. A sterile 5-μL Hamilton syringe with a 26 gauge needle was inserted to a depth of 3.0 mm from the skull surface and withdrawn by 0.5 mm to inject 3 × 10^3^ U87MG or TPC8 cells in a volume of 3 μL. The injection rate was set to 1 μL/min. After the implantation of the tumor cells, the needle was left in place for 5 min to prevent reflux. The needle was then completely withdrawn from the brain over the course of 4 min and the skin was sutured. Treatments were started 5 days after cell injection when no luciferase activity was intracranially detectable. At this time, animals were randomized to receive vehicle, Bevacizumab (4 mg/kg iv every 4 days), or UniPR1331 (30 mg/kg po qd), or combinations of Bevacizumab with UniPR1331. Weekly, animals were tested for bioluminescence assay. The time in which a visible bioluminescence lesion defined the disease-free survival (DFS). Repeated bioluminescence assays were planned in order to have also data on tumor progression. Bioluminescence imaging (BLI) was performed by using the Alliance Mini HD6 (UVItec Limited, Cambridge, United Kingdom) after injection ip with 150 μg/g D-luciferin (Synchem UG & Co. Altenburg, Germany) in pre-anesthetized animals. Duration of treatments was of 30 days at the end of which a period of follow-up was planned. Mice were euthanized when they displayed neurological signs (e.g., altered gait, tremors/seizures, lethargy) or weight loss of 20% or greater of pre-surgical weight. Follow-up provide us data on overall survival defined as the time (days) in which the animal into account showed the distress signs considered (euthanized time). Brains were collected, fixed with 4% paraformaldehyde and paraffin embedded.

### Histological analysis

Immunohistochemical evaluation were performed for EphA2 and p-Tyr EphA2 in U87MG xenografts. Indirect immunoperoxidase staining of tumor xenografts samples was performed on paraffin-embedded tissue sections (4 μm) following standard conditions. The evaluations were recorded as the percentage of positively stained tumor cells in each of three intensity categories. A consensus judgment as indicated in our previous report [51] was adopted as to the proper immunohistochemical score of the tumors based on the strength of antigen expression: negative (score 0), weak staining score 1), moderated staining (score 2), or strong staining (score 3). In each category, the percentage of positively stained tumor cells was assessed by scoring at least 1000 adjacent cells in the area with the highest density of antigen-positive cells. Slides were analyzed separately by CF, GLG and RS. Cytoplasmic/membrane staining intensity was graded as follow: 0= negative; 1= less of 10% of positive cells; 2= positive cells in a range of 10-50% and 3= more than 50% positive cells. Staining Index (SI), an indicator of overall expression levels, ranged between 0 and 9 with an SI ≤ 4 indicating a low expression. Ki67 labeling index was determined by counting 500 cells at 100X and determining the percentage of cells staining positively for Ki67. Apoptosis was determined by using the TACS TdT *in situ* TACS Blue Label kit (code 4811-30-K; R&D Systems, Inc. Minneapolis, MN). Apoptosis was measured as the percentage of tunnel positive cells measured on five random fields (400 X). Tumor microvessels were counted at ×400 in five arbitrarily selected fields and the data were presented as number of CD31+ microvessels/×100 microscopic field for each group. The presence of red cells in tumor tissue and in blood vessels as well as the presence of micro-thrombi and bleeding zones was demonstrated by Martius yellow-brilliant crystal scarlet blue technique.

### MiRNA isolation and real-time quantitative PCR analysis

Total RNA, miRNAs enriched, was extracted from the glioblastoma cell lines at baseline and after treatment with UniPR1331 compound, using the AllPrep DNA/RNA/miRNA Universal kit (Qiagen, Germany) according to the manufacturer's instructions. RNA quantification and purity was measured with Qubit Fluorometer v3.0 (Thermo Scientific). Seven-hundred ng of RNA was subjected to retro-transcription (RT) by using the TaqMan® Micro-RNA Reverse Transcription kit (Thermo Fisher Scientific, CA, USA) and analyzed for miRNAs’ expression levels with the TaqMan® human microRNA array A and array B v3.0 (Thermo Fisher Scientific). The two arrays consist in two 384-well microfluidic cards containing primers and probes for 754 different human miRNAs in addition to 3 small nucleolar RNAs that function as endogenous controls for data normalization. Six μl of each RT reaction was combined with 450 μ of TaqMan® Universal PCR Master Mix, No AmpErase® UNG, 2 ✕ (Thermo Fisher Scientific) and 444 μl of nuclease-free water. One hundred μl of the sample/master mix for each multiplex pool were loaded into fill reservoirs of the microfluidic cards; the array was then centrifuged and mechanically sealed with the Applied Biosystems sealer device. Quantitative PCR was carried out on an ViiA-7 system thermo-cycler (Thermo Fisher Scientific) using the manufacturer's recommended cycling conditions. Data analysis was performed by using the SDS software version 2.3 (Thermo Fisher Scientific) and the baseline and threshold were automatically set. The average levels of miRNAs expression in all samples were normalized relative to the average amounts of U6 small nuclear RNA (U6 snRNA). The ΔΔCt comparative method was applied to measure the miRNAs that are differentially expressed between the UniPR1331-treated and untreated cells. We considered Relative Quantification values RQ ≥2 or RQ≤ 0.5 as up- and down-regulation cut-off, respectively.

### Computational analysis

MiRNAs potentially influencing the angiogenesis reduction were identified by selecting validated and predicted miRNAs targeting angiogenesis-related genes through the miRWalk 2.0 bioinformatic tool [52]. The miRWalk Validate Module was first queried to identify UniPR1331-induced miRNAs having experimentally verified targets genes in “positive regulation of angiogenesis” Gene Ontology term (GO:0045766) database and UniPR1331-reduced miRNAs with targets in “negative regulation of angiogenesis” term (GO:0016525) database and then to select miRNAs also involved in glioblastoma disease, according to the Disease Ontology DOID:3068. The experimentally validated interaction between UniPR1331-deregulated miRNAs and genes involved in GO:0045766, GO:0016525 and/or DOID:3068 ontologies were included in Cytoscape 3.4 [53] tools to provide a graphical representation of miRNAs-targets interactions. For predictive enrichment analysis of UniPR1331-deregulated miRNAs in angiogenesis functional GO:0045766 or GO:0016525 pathways, miRNAs predicted with a p-value <0.01 to bind their targets were considered potentially involved in reduction of angiogenesis and the relative genes as highly probable targets transcript. The Venny2.0 tool was used to draw the Venn diagram [54].

### MiRWalk algorithm

The Prediction Module of the miRWalk algorithm is a comparative platforms that predicts miRNA binding sites based on a comparison of data from 13 established miRNA-target prediction tools: DIANA-microTv4.0, DIANA-microT-CDS, miRanda-rel2010, mirBridge, miRDB4.0, miRmap, miRNAMap, doRiNA i.e., PicTar2, PITA, RNA22v2, RNAhybrid2.1, and Targetscan6.2. The miRWalk Validated Module documents experimentally verified miRNA-target interaction information collected via an automated text-mining search and data from existing resources (miRTarBase, PhenomiR, miR2Disease and HMDD) offer such information. MiRNAs potentially influencing the angiogenesis reduction were identified by selecting validated and predicted miRNAs targeting angiogenesis-related genes through miRWalk Predicted Module and miRWalk Validate Module. For the enrichment of miRNAs in angiogenesis functional pathways, the miRWalk Prediction Module was used against Gene Ontology Biological Process “positive regulation of angiogenesis” term (GO:0045766 for UniPR1331-induced miRNAs and “negative regulation of angiogenesis” term (GO:0016525) for UniPR1331-repressed miRNAs, that include respectively 78 and 51 genes. The list of miRNAs predicted by miRWalk algorithm to interact with at least one of genes associated to GO:0045766 and GO:0016525 ontologies was compared to the list of UniPR1331-induced miRNAs or UniPR1331-repressed miRNAs, respectively. The resulting UniPR1331-deregulated miRNAs targeting GO:0045766 or GO:0016525 related genes and having the interaction p-value <0.01 were considered potentially involved in reduction of angiogenesis and the relative genes as highly probable targets. The miRWalk Validate Module was queried to identify UniPR1331-deregulated miRNAs having experimentally verified targets genes in GO:0045766 or GO:0016525 ontologies and then to select miRNAs also involved in glioblastoma disease, according to the Disease Ontology DOID:3068.

### Statistical details

Continuous variables were summarized as mean and standard deviation (SEM) or as median and 95% CI. For continuous variables not normally distributed, statistical comparisons between control and treated groups were established by carrying out the Kruskal-Wallis Tests and Dwass-Steel-Chritchlow-Fligner method. For continuous variables normally distributed, statistical comparisons between control and treated groups were established by carrying out one- or two-way ANOVA test (further details in the panel's legend). Significant differentially expressed miRNAs were detected through Student paired t-test. TTP was analyzed by Kaplan-Meier curves and Gehan's generalized Wilcoxon test. The use of Kaplan Meier Curves allow investigators to study a wide range of planned pre-specific events or endpoints such as death, disappearance or progression of a tumor also in preclinical settings. When more than two survival curves were compared the Log rank test for trend was used. This tests the probability that there is a trend in survival scores across the groups. All tests were two-sided and were determined by Monte Carlo significance. P values <0.05 were considered statistically significant. MedCalc was used as a complete statistical program. We analyzed Kaplan Meier curves [48] in term of hazard ratios (HR), an expression of the hazard or chance of events occurring in the treatment arm as a ratio of the hazard of the events occurring in the control arm.

## SUPPLEMENTARY MATERIALS FIGURES AND TABLES


