# Supplementary material for: UniPR1331, a small molecule targeting Eph/ephrin interaction, prolongs survival in glioblastoma and potentiates the effect of antiangiogenic therapy in mice
Source: Oncotarget. 2018 May 11;9(36):24347–63. doi: 10.18632/oncotarget.25272 (PMC5966254; doi:10.18632/oncotarget.25272)
Supplement: Supplementary file 1 [file oncotarget-09-24347-s001.pdf]

UniPR1331, a small molecule targeting Eph/ephrin interaction, prolongs survival in glioblastoma and potentiates the effect of antiangiogenic therapy in mice

SUPPLEMENTARY MATERIALS

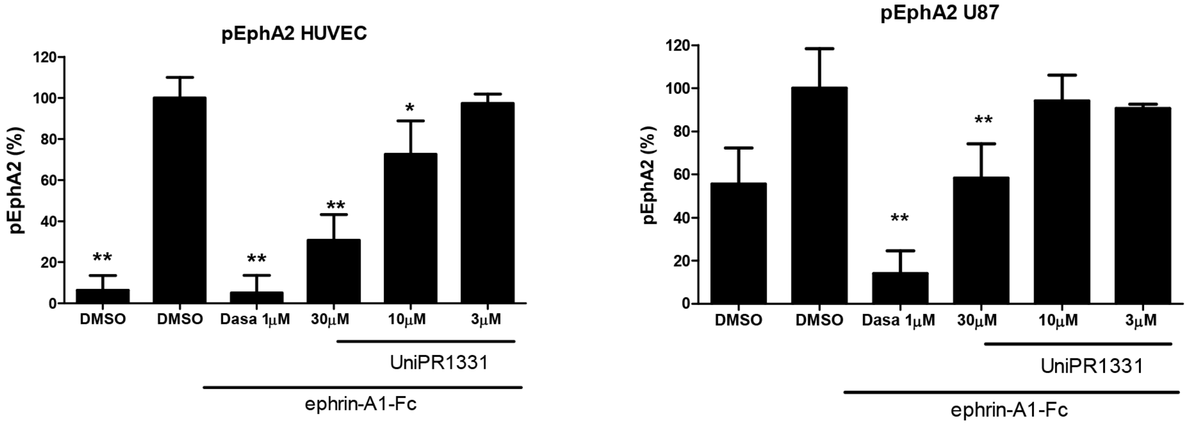

Supplementary Figure 1: EphA2 phosphorylation *in vitro* on HUVE and U87MG cells. UniPR1331 dose-dependently inhibited EphA2 activation induced by ephrin-A1-Fc in U87MG and HUVE cells. Cells were pretreated for 20 minutes with 0.3% DMSO, or the indicated concentrations (µM) of compounds, and then stimulated for 20 minutes with ephrin-A1-Fc. Phospho-EphA2 levels are relative to ephrin-A1-Fc+DMSO. Data are the means of at least three independent experiments ± st. dev. One-way ANOVA followed by Dunnet's post-test was performed to compare ephrin-A1-Fc+DMSO to all the other columns. *, $p<0.05$, **, $p<0.01$.

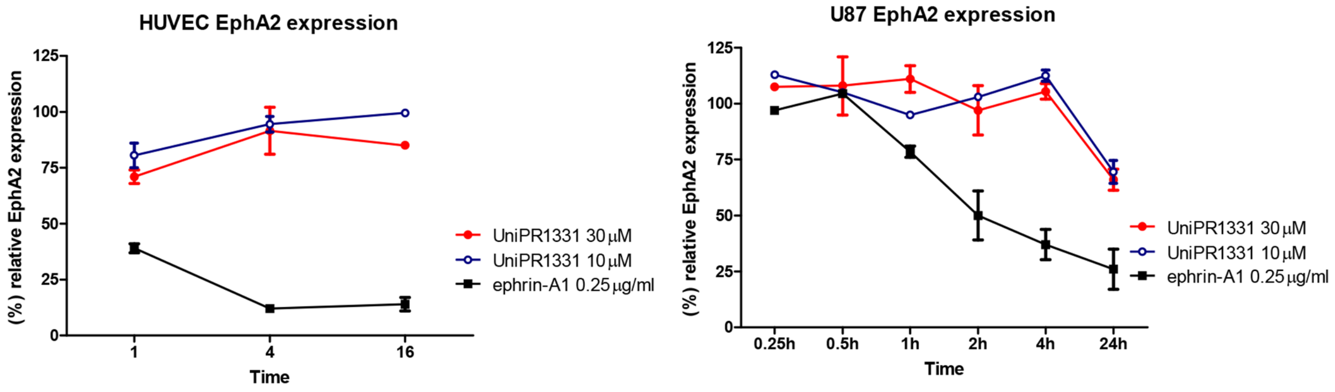

Supplementary Figure 2: EphA2 expression *in vitro* or UniPR1331 incubation on HUVE and U87MG cells. Ephrin-A1 time-dependently reduced EphA2 expression whereas UniPR1331 reduced its expression only after 24h incubation in U87MG cells. Cells were treated for the indicated time with 0.3% DMSO, 0.25µg/ml ephrin-A1 or 10/30µM UniPR1331. Data were normalized for each time point to DMSO as 100% EphA2 expression. Experiments were performed via ELISA using the product DYC3035-2 of R&D systems following manufacturer's procedures.

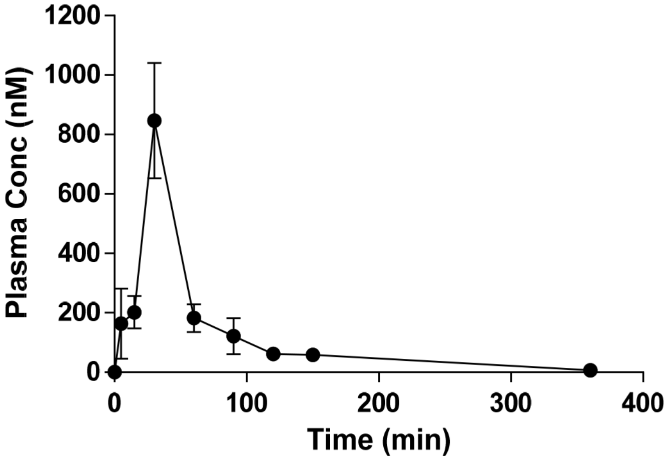

Supplementary Figure 3: Pharmacokinetic. Plasma profile of UniPR1331. Plasma concentrations (nM) of the compounds over 360 minutes time course after a single administration in male mice (30 mg/kg, os) are reported as obtained from HPLC/MS analysis. Data are the means of at least three independent experiments ± SEM. Brain concentrations (pmol/g) of UniPR1331 over 360 minutes time course after a single administration in male mice (30 mg/kg, os) are reported as obtained from HPLC/MS analysis. Data are the means of three independent experiments ± SEM

Time (min)	UniPR1331 Brain Concentration (pmol/g)[a]	SEM
0	0	-
15	5.5	1.0
30	98	17.8
60	33	9.0
360	N. D.[b]	-

[a] Amoung found for g of tissue. pmol/g ≈ nM. Not Detected.

Supplementary Table 1: UniPR1331 activity on several targets

Target	Study	% Inhibition or activation	Concentration
EphA2 inhibition	LANCE	Inactive	10 microMolar
VEGFR inhibition	LANCE	Inactive	10 microMolar
K ATP channel	Binding	Inactive	10 microMolar
PDGFR	Binding	Inactive	10 microMolar
TGF beta	Binding	Inactive	10 microMolar
ICAM-1	Functional	Inactive	10 microMolar
LXR alpha agonist	Functional	Inactive	10 microMolar
LXR alpha antagonist	Functional	Inactive	10 microMolar
LXR beta agonist	Functional	Inactive	10 microMolar
LXR beta antagonist	Functional	Inactive	10 microMolar
EGFR agonist	Functional	Inactive	10 microMolar
EGFR antagonist	Functional	Inactive	10 microMolar
FGFR antagonist	Functional	Inactive	10 microMolar
PPAR gamma agonist	Functional	Inactive	10 microMolar
PPAR gamma antagonist	Functional	Inactive	10 microMolar
GLP1 agonist	Functional	Inactive	10 microMolar
GLP1 antagonist	Functional	Inactive	10 microMolar
TGR5 agonist	Functional	Inactive	10 microMolar
DPP-IV	Enzymatic	Inactive	10 microMolar

Supplementary Table 2: Biochemical chemistry and histological analysis

	Treated	Control
Leucocytes (10^3/µl)	6.6±0.9	5.0±1.9
Neutrophils (10^3/µl)	2±0.2	1.6±0.4
Lymphocytes (10^3/µl)	4.3±0.7	3.3±1.4
Monocytes (10^3/µl)	0.18±0.06	0.14±0.16
Eosinophils (10^3/µl)	0.04±0.02	0±0
Basophils (10^3/µl)	0.02±0.01	0.02±0.02
Erythrocytes (10^6/µl)	8.9±0.1	8.9±0.1
Emoglobin (g/dl)	14.2±0.2	12.1±2.1
Hematocrit (%)	40.6±0.7	40.2±0.6
Mean cell volume (fl)	45.3±0.2	45.4±0.3
Hb cell mean cont (pG)	15.9±0.1	16.0±0.1
Platelets (10^3/µl)	207±87	714±171
Mean platelet volume (fl)	5.5±0.2	5.1±0.2
Glucose (mg/dl)	154±8	151±10
Urea-N (mg/dl)	53±3	51±8
Cholesterol (mg/dl)	73.6±3.5	64±4
Total protein (g/dl)	4.7±0.1	4.6±0.2
ALT (IU)	53.2±3.8	44.8±6.8
Creatinine (mg/dl)	0.454±0.03	0.508±0.03
AST/GOT (UI)	125±8	117±12

Observation of mice treated with UniPR1331 did not result in any alteration in eyes, mucous membranes, occurrence of secretions and excretions and autonomic activity (e.g., lacrimation, piloerection, unusual respiratory pattern), changes in gait, posture and response to handling as well as the presence of clonic or tonic movements, stereotypes (e.g., excessive grooming, repetitive circling) or bizarre behavior (e.g., self-mutilation, walking backwards). No other major neurological difference were detected at observation. Hematology and clinical biochemistry analysis reported a low platelet count of 207x10^3/µl but no clinically significant difference in other studied parameters (blood count, Hb, hematocrit, glucose, total cholesterol, urea, creatinine, total protein and albumin, aspartate aminotransferase, alkaline phosphatase) were found. Rare focus of cellular injury with increased mitoses and nuclear alterations were observed in liver; adaptive changes (cellular swollen and cellular hypertrophy) were observed in both liver and kidney. No alteration was found in testis, heart, gastrointestinal tract, lung and CNS.

Supplementary Table 3: Antitumor activity of UniPR1331 alone or in combination with Bevacizumab in U87MG xenografts

*p<0.05 vs controls ** p<0.005 (beva vs Uni)	Weight of mice (gr +/- SE)	Tumor weight (mg +/- SE)	TTP (days +/_ SE)	Vessel count (+/- SE)	Necrotic areas (% +/- SE)	Ki67 (% +/- SE)	Apoptosis (% +/- SE)
Saline	24.5 ± 0.6	709 ± 145	9.9 ± 0.9	23.2± 2.3	25.0 ± 3.5	44.6 ± 3.5	< 2
UniPR1331	26.0 ± 0.4	372 ± 72*	15.8± 0.9** and **	5.5 ± 0.2**	16.5 ± 3.5£	14.5 ± 2.5**	8.5 ± 1.1
Bevacizumab	24.8 ± 0.55	360± 94*	13.8± 0.5**, *	6.4±0.5**, £	27.0 ± 3.0*	22.6 ± 1.9**	< 2
UniPR1331 + Bevacizumab	25.5 ± 0.3	179 ± 59**, £	22.2± 0.8**	1.3 ± 0.2**	35.5 ± 3.5*, **	10.7 ± 1.5**	34.5 ± 3.5

(1) no statistical significance in the weight of mice.
(2) *p<0.005 vs Saline and **p<0.0001 vs Saline no statistical significance between UniPR1331 and Bevacizumab; £ p<0.05 in the comparison between combination vs single treatments;
(3) **p<0.0001 vs Saline and in the comparison between combination vs single treatments; *p<0.005 in the comparison Bevacizumab vs UniPR1331.
(4) **p<0.0001 vs Saline and combination vs single treatments; £P<0.05 Bevacizumab vs UniPR1331;
(5) *p<0.005 and £p<0.05 vs saline; **p<0.0001 combination vs UniPR1331, °p<0.005 combination vs Bevacizumab;
(6) **p<0.0001 for all comparisons except £p<0.05 combination vs UniPR1331.
(7) ** p<0.001 vs saline.

Supplementary Table 4: Comparison of Kaplan Meier curves in U87MG xenografts

Comparison	Hazard ratio	Confidence interval	Significance
Vehicle vs UniPR1331	3.84	1.20 to 9.82	P < 0.0005
Vehicle vs Bevacizumab	3.79	1.29 to 11.13	P<0.001
UniPR1331 vs Bevacizumab	1.86	0.74 to 4.67	P = 0.0631
Vehicle vs UniPR1331 + Bevacizumab	4.70	1.88 to 14.86	P<0.001
UniPR1331 vs UniPR1331 + Bevacizumab	3.45	1.21 to 9.87	P < 0.001
Bevacizumab vs UniPR1331 + Bevacizumab	4.24	1.89 to 12.93	P<0.001

Supplementary Table 5: Antitumor activity of UniPR1331 alone or in combination with Bevacizumab in U251MG xenografts

Drug	Weight of mice (gr +/- SE)*	Tumor weight (mg +/- SE)	TTP (days +/_ SE)	Vessel count (+/- SE)	Necrotic areas (% +/- SE)	Ki67 (% +/- SE)	Apoptosis (% +/- SE)
Saline	24.6 ± 0.8	839± 175	10.0 ± 3.0	33.2 ± 2.0	18.0 ± 2.5	38.4 ± 5.0	< 2
UniPR1331	25.4 ± 0.5	311 ± 60	13.6± 3.7	11.6 ± 3.7**	19.6 ± 1.8	15.4 ± 1.5	8.5 ± 2.6**
Bevacizumab	24.8 ± 0.3	345± 119	14.4± 1.6£	9.5 ± 0.8 **	20.4 ± 2.7	24.5 ± 2.6	< 2
UniPR1331 + Bevacizumab	23.5 ± 0.4	157 ± 47	20.6± 4.9*£	4.0 ± 4**, *	17.7 ± 1.4	8.5 ± 1.5	20.5 ± 4.5**

(1) no statistical significance in the weight of mice.
(2) * p<0.005 vs saline; £p<0.05 in the comparison between combination vs single treatments;
(3) *P<0.005 vs saline and in the comparison between combination vs single treatments; *p<0.005 in the comparison Bevacizumab vs UniPR1331 and in comparison with combination and single treatments.
(4) **p<0.0001 vs Saline and combination vs Bevacizumab; *p<0.005 combination vs Bevacizumab and NS Bevacizumab vs UniPR1331;
(5) no statistical differences between groups.
(6) **p<0.0001 for all comparisons.
(7) ** p<0.001 vs saline.

Supplementary Table 6: Comparison of Kaplan-Meier curves in U251MG xenografts

Comparison	Hazard ratio	Confidence interval	Significance
Vehicle vs UniPR1331	2.41	0.88 to 8.57	P=0.285
Vehicle vs Bevacizumab	3.01	1.09 to 8.31	P<0.05
UniPR1331 vs Bevacizumab	0.90	0.37 to 2.17	P = 0.7585
Vehicle vs UniPR1331 + Bevacizumab	3.95	1.33 to 11.76	P<0.001
UniPR1331 vs UniPR1331 + Bevacizumab	2.93	1.47 to 8.00	P<0.05
Bevacizumab vs UniPR1331 + Bevacizumab	2.86	1.35 to 7.77	P<0.05

Supplementary Table 7: UniPR1331-deregulated miRNAs and targeted genes potentially involved in reduction of angiogenesis

	miRNAs[a]	RQ	Enrichment p value[b]	Putative number of targeted genes[c]	High potentially target genes[d]
**UniPR-induced miRNAs**	hsa-miR-1208	2.021	1.94E-21	78	*CHI3L1*
	hsa-miR-1254	3.396	1.76E-17	78	*CX3CL1*
	hsa-miR-183-3p	15.021	1.16E-28	78	*CHRNA7, NTRK1, TWIST1*
	hsa-miR-30c-1-3p	8.097	7.96E-16	78	*ADM2 RRAS*
	hsa-miR-329-3p	12.024	1.49E-27	78	*HIPK1, TWIST1*
	hsa-miR-34b-3p	4.038	3.55E-32	78	*IL1A*
	hsa-miR-543	2.850	3.62E-22	78	*C5*
**UniPR-reduced miRNAs**	hsa-miR-1226-5p	0.026	8.41E-11	51	*ROCK1, PML*
	hsa-miR-1270	0.028	1.84E-12	51	*SPINK5*
	hsa-miR-1298-5p	0.363	3.30E-17	51	*PML*
	hsa-miR-1303	0.206	4.28E-13	51	*ANGPT4, NF1*
	hsa-miR-130a-5p	0.029	2.99E-17	51	*HRG, THBS4*
	hsa-miR-206	0.400	2.58E-18	51	*CCL2*
	hsa-miR-326	0.018	1.36E-18	51	*PDE3B, PML*
	hsa-miR-338-5p	0.405	2.21E-23	51	*TNMD*
	hsa-miR-432-3p	0.067	3.67E-20	51	*PML*
	hsa-miR-501-3p	0.012	2.24E-17	47	*PF4*
	hsa-miR-520c-3p	0.033	1.48E-19	51	*FASLG, GTF2I*
	hsa-miR-545-3p	0.284	2.34E-21	51	*TNMD*
	hsa-miR-548b-5p	0.031	2.56E-20	51	*SPINK5, KRIT1, NF1, STAT1, RIT1K*
	hsa-miR-593-3p	0.005	1.24E-14	51	*NPR1*
	hsa-miR-659-3p	0.323	3.04E-14	51	*FASLG, THBS4*
	hsa-miR-7-2-3p	0.294	4.79E-20	51	*GTF2I*
	hsa-miR-889-3p	0.343	0.000463174	23	*SPINK5*
	hsa-miR-941	0.134	1.90E-09	31	*COL4A3*
	hsa-miR-942-5p	0.256	1.30E-12	51	*COL4A3, THBS4*

a. UniPR-deregulated miRNAs predicted to have significant ($p<0.01$) interactions with angiogenesis–related genes according the miRWalk algorithm; b. Fischer p value calculated by miRWalk tool in the prediction analysis of miRNAs enrichment for their binding sites within the genes on GO:0045766 and GO:0016525 ontologies; c. Putative number of targeted genes in the GO:0045766 and GO:0016525 annotations for UNIPR-induced miRNAs and UNIPR- reduced miRNAs, respectively; d. High significantly ($p<0.01$) predicted targeted genes, according to miRWalk algorithm. RQ, relative quantification.

Supplementary Table 8: UniPR1331-induced miRNAs with experimental validated targeted genes on glioblastoma DOID:3068 ontology

UniPR-induced miRNAs	miRNAs Validated targeted genes
hsa-miR-101-5p	*ATM*
hsa-miR-1179	*ADAMTS4, CDK6, HMGB1, SOD2, WEE1*
hsa-miR-1208	*FYN, NFATC2*
hsa-miR-1254	*ERBB2, PKM*
hsa-miR-1255b-5p	*, EPHA4, EPHA2, MAPK1, MDM2, PIN1*
hsa-miR-137	*AURKA, CDK6, KIT, MET, MTDH, PTGS2, TLR3*
hsa-miR-15a-3p	*VAV3*
hsa-miR-183-3p	*CDK6, NAMPT, PROM1, SOD2*
hsa-miR-190b	*IGF1, KLF6*
hsa-miR-26a-2-3p	*BIRC5, PRKCH, PTEN*
hsa-miR-30c-1-3p	*ADAMTS4, IGF1, PIN1, POLR3K, PPIA, POLR3K, TNFRSF10B*
hsa-miR-329-3p	*ADAMTS4, CDK6, CTGF, CXCL12, ELK1, IGF1R, PIK3CG, PIK3R1, SOD2, TWIST1, VEGFA*
hsa-miR-337-3p	*CLDN1, FPR1, RICTOR, STAT3*
hsa-miR-34b-3p	*BCL2, CCND1, CDK6, CREB1, MET, MYC, NOTCH1, SNAI1, SOX2, VEGFA, WEE1*
hsa-miR-363-5p	*IGF1R, PKM, VEGFA*
hsa-miR-376a-5p	*CREB1, PTEN*
hsa-miR-449b-5p	*CDK4, CDK6, CDKN1B, MET, VAV3*
hsa-miR-487b-3p	*MYC, KRAS*
hsa-miR-502-3p	*CDK6, SOD2*
hsa-miR-543	*ATM, HMGA2, PTK2, PTEN, TWIST1*
hsa-miR-616-3p	*CDK4*

Supplementary Table 9: UNIPR-reduced miRNAs with experimental validated targeted genes on glioblastoma DOID:3068 ontology

UniPR -reduced miRNAs	Validated targeted genes
hsa-miR-1226-5p	*AURKA, CREB1, VAV3*
hsa-miR-125b-1-3p	*IGF1R, S1PR, SOD2,*
hsa-miR-1270	*MDM2, MTDH*
hsa-miR-1291	*ABCC1*
hsa-miR-1298-5p	*CDK6, EDN1, TAPBP*
hsa-miR-1303	*CDKN1B, CDK6, CYR61, HOXA10, MET, PHF20*
hsa-miR-130a-5p	*VAV3, SOD2, WEE1*
hsa-miR-144-5p	*SOD2*
hsa-miR-199a-3p	*AKT1, MAPK1, IGF1, HGF, MAPK8, MET, MTOR, PKM, PTGS2, SOD2*
hsa-miR-206	*BCL2, CDK4, CCND1, GJA1, KRAS, IGF1R, MET, SMARCB1, WEE1*
hsa-miR-26a-1-3p	*BIRC5, PRKCH, PTEN*
hsa-miR-32-5p	*ADAM10, AURKA, BCL2L11, HMGA2, ITGA6, MDM2, TLR3, TRIO, PTEN*
hsa-miR-326	*AKT1, CCND1, ERBB2, GLI1, KRAS, NOTCH1, PKM, XRCC6*
hsa-miR-338-5p	*CLDN1, CTGF, HOXA10, LRP1*
hsa-miR-449a	*BCL2, CCND1, CDKN1, CDK4, CDK6, MYC, MET, NOTCH1, VAV3,*
hsa-miR-501-3p	*CDK6, SOD2*
hsa-miR-520c-3p	*APP, CREB1, IGF1R, HMGB1, MTOR, TNFRSF10B, WEE1*
hsa-miR-545-3p	*CDK4, CCND1, XRCC6, SOD2, LRP1, WEE1*
hsa-miR-548b-5p	*CDK4, INHBA, XRCC6, CHEK2, POLR1B*
hsa-miR-549a	*MTDH, KIT*
hsa-miR-551b-5p	*APP, CDK6, ERCC1, IGF1R, SOD2*
hsa-miR-572	*ATM, CDKN1A*
hsa-miR-584-5p	*FYN, MAPK1*
hsa-miR-593-3p	*CCND1, ERBB2, FHL2, SOD2*
hsa-miR-638	*SOD2, SOX2*
hsa-miR-639	*CDKN1A*
hsa-miR-659-3p	*PYGO2*
hsa-miR-7-2-3p	*KLF6, PHLPP1, RHOB, TWIST1, VEGFA*
hsa-miR-744-3p	*CCR2, CDK4*
hsa-miR-766-3p	*BAX, CDK4, ERCC1, GAPDH, MAPK1, MDM2, PHF3, PKM, POLR3K, VAV3, XRCC6*
hsa-miR-767-5p	*CASP8, HLA-A, MDM2, MMP2*
hsa-miR-889-3p	*ABCG2, HIF1A*
hsa-miR-92a-1-5p	*FAS, MYC, PTEN, TNFRSF12A, TP53*
hsa-miR-941	*XRCC6*
hsa-miR-942-5p	*CDK6, CDKN1A, GALNT13, PHF3, PTPN11*
